# Effects of Fermented Green Tea Waste Extract Gels on Oxidative Damage in Short-Term Passive Smoking Mice

**DOI:** 10.3390/gels8080461

**Published:** 2022-07-22

**Authors:** Jiangwen Liu, Yijun Wang, Lei Sun, Dongfeng Guo, Xuefeng Wu, Dongdong Mu, Xingjiang Li

**Affiliations:** 1 School of Food and Bioengineering, Hefei University of Technology, Hefei 230009, China; liujiangwen1213@mail.hfut.edu.cn (J.L.); wuxuefeng@hfut.edu.cn (X.W.); d.mu@hfut.edu.cn (D.M.); 2 State Key Laboratory of Tea Plant Biology and Utilization, Anhui Agricultural University, Hefei 230036, China; yijun@ahau.edu.cn; 3 Sinopharm Health Biotechnology (Huangshan) Co., Ltd., Huangshan 245061, China; sunlei_hfut@hotmail.com; 4 Technology Center of Anhui China Tobacco Industry Co., Ltd., Hefei 230088, China; guodongfeng-hfut@hotmail.com

**Keywords:** fermented green tea waste extract gels, gel properties, oxidative damage, short-term, passive smoking

## Abstract

Passive smoking is extensively studied because of its harmfulness to human health. In this study, the effects of fermented green tea waste extract gels (GTEG) on oxidative damage in mice exposed to short-term cigarette smoke (CS) were investigated. The GTEG is prepared from green tea waste extract and microbial transglutaminase (MTGase). The lung injury model of mice was established through passive smoking for 5 days. The experimental results revealed the following findings. (1) The GTEG induced by MTGase has obvious gel properties; (2) GTEG has strong biological activity and antioxidant properties in vitro; (3) The passive smoking model was established successfully; specifically, the lung tissue of the model mice exhibited inflammatory symptoms, oxidative stress response appeared in their bodies, and their inflammatory indicators increased; (4) Compared with the passive smoking model group, the mice, which were exposed to CS and received GTEG treatment, exhibited increased food intake and body weight; increased total superoxide dismutase and glutathione peroxidase activity in serum; significant decreases (*p* < 0.05) in the content levels of the inflammatory factors malondialdehyde, interleukin (IL)-6, and tumor necrosis factor α (TNF-α); and inhibited expression of IL-6, IL-33, TNF-α, and IL-1β inflammatory genes. The results indicated that taking GTEG can relieve the oxidative stress injury of mice caused by short-term CS and has antioxidant properties.

## 1. Introduction

Smoking is a notorious health risk factor that contributes to the occurrence of cardiovascular and respiratory diseases and several types of cancer. Smoking kills more than 8 million people annually according to the 2020 data published by the World Health Organization [1]. In today’s social environment, habits, occupation, and other factors make it difficult for people to avoid active or passive smoking, and passive smoking can considerably threaten human health. Cigarette smoke (CS) consists of the mainstream smoke exhaled by smokers and the side-stream smoke emitted by burning tobacco [2]. Numerous studies have reported that CS is a key source of various carcinogenic volatile organic compounds [3]. CS contains numerous oxidants and pro-oxidants, which turn to be reactive oxygen species (ROS) and active nitrogen (RNS) when they enter the human body. Excessive ROS and RNS produce oxidative stress, leading to oxidative damage to lipids, DNA, and proteins [4]. The increased oxidative stress injury induced by CS is regarded as the first step in the pathogenesis of abnormal lung function, obesity, neurobehavioral disorders, and cardiovascular diseases [5], which are huge threats to human health. Therefore, passive smoking is a topic requiring further investigation.

In recent years, studies have discovered that the strong antioxidant properties of green tea can prevent and treat chronic diseases caused by CS exposure [6] and help reduce the oxidative damage in the body. As one of three major global beverages, green tea is favored by people not only for its taste, but also for its various functions that benefit physical and mental health. Green tea is rich in tea polyphenols, of which epigallocatechin gallate (EGCG) is a main component. EGCG is an excellent natural product that is beneficial to wound healing [7], which can act on the gastric adenocarcinoma cell line AGS. The AGS cell line can be stimulated by chemical attractants to release IL-1β, which can cause inflammation. Tea extract can considerably reduce the release of IL-1β, indicating that green tea extract (GTE) can reduce inflammation in the body [8]. The active ingredients in green tea also substantially reduce the DNA damage in upper respiratory tract cells induced by CS condensates [9]. CS exposure considerably reduces the activity levels of glutathione peroxidase (GSH-Px) in the serum of mice and increases the expression of malondialdehyde (MDA), tumor necrosis factor α (TNF-α), interleukin (IL)-6, IL-8, and IL-1β in serum. The adverse reactions of tea and tea particles can be alleviated [10]. In conclusion, green tea and GTE can effectively relieve oxidative stress, inflammation, and the other adverse reactions induced by smoking. The aforementioned studies are based on long-term smoke exposure; however, no systematic study has conducted an artificial stimulation of a short-term passive smoking environment to explore the effects of fermented green tea waste extract (GTEG) on short-term passive smoking injury in mice. People who are exposed to passive smoking tend to exhibit the characteristics of short-term CS inhalation.

Microbial transglutaminase (MTGase) is currently used to improve the gelling properties of soybean protein, whey protein, peanut protein and other proteins, MTGase is an enzyme that action between glutamine and lysine protein led to a cross-linking and polymerizing effect by ε-(γ-glutamyl) lysine bonds [11]. In addition, MTGase improved the gel strength and thermal stability of plant proteins. Priya et al. [12] synthesized Gel-oxGTp-phen self-healing hydrogel from green tea extract and gelatin with flexible and self-repairing properties, and showed good biodegradability in urea solution, phosphate buffer and acidic/alkaline solution [12]. In this study, we prepared GTEG with green tea waste extract and MTGase, and studied the effect of GTEG on the oxidative damage response in vivo. Mice were exposed to smoke for a short period (5 days). The food intake, body weight, and lung morphology characteristics of mice were examined; furthermore, the changes in their serum levels of oxidative stress and their expression of IL-6, IL-33, lung tissue TNF-α, and other inflammatory genes were analyzed. The effect of GTEG on the short-term passive smoking lung injury in mice that received oxidative stress intervention was analyzed. Our study would inspire the future application of GTEG as an anti-inflammatory antioxidant.

## 2. Materials and Methods

### 2.1. Materials

The main chemicals used in the present study are as follows: Green tea waste was provided by Sinopharm Health Biotechnology (Huangshan, China); green tea fermentation strain Eurotium cristatum, provided by China Center of Industrial Culture Collection; cigarettes standard were provided by China Tobacco Anhui Industry; commercial MTG was provided by Jiangsu Yiming Biological; Folin’s phenol reagent, aluminum trichloride, analytical grade concentrated sulfuric acid, anthrone, Coomassie Brilliant Blue, ninhydrin, xylene, anhydrous ethanol, anhydrous methanol, and acetic acid were provided by Shanghai Yuanye Bio-Technology; RNA extract, nuclease-free water, RT First Strand cDNA Synthesis Kit, 2 × SYBR Green qPCR Master Mix (no ROX), and primers were purchased from Wuhan Servicebio Technology; total superoxide dismutase (T-SOD) test kit, MDA test kit, GSH-Px test kit, mouse IL-6 ELISA kit, and mouse tumor necrosis factor α (TNF-α) ELISA kit were purchased from ABclonal Biotechnology Company (Woburn, MA, USA).

### 2.2. Experimental Instruments

The following experimental instruments were used in the present study: Agilent LC 1100 liquid chromatography system and chromatographic column (Agilent C18 column), provided by Agilent Technologies Co., Ltd., Beijing, China; inverted microscope, provided by Carl Zeiss Co., Ltd., Suzhou, China; ultraviolet-visible spectrophotometer, provided by Shanghai yuanxie Instrument Co., Ltd., Shanghai, China; Nicolet 6700 FTIR spectrometer, provided by Thermo Nicolet Co., Ltd., Madison, WI, USA; TA-XT plus texture analyzer, provided by Stable Micro Systems Co. Ltd., Godalming, UK; Quanta250FEG scanning electron microscope, provided by FEI Company, State of Oregon; DHR-3 rheometer, provided by TA Instruments, Leatherhead, UK.

### 2.3. Green Tea Waste Fermentation and Production of GTEG Crude Liquor

**Figure 1:**
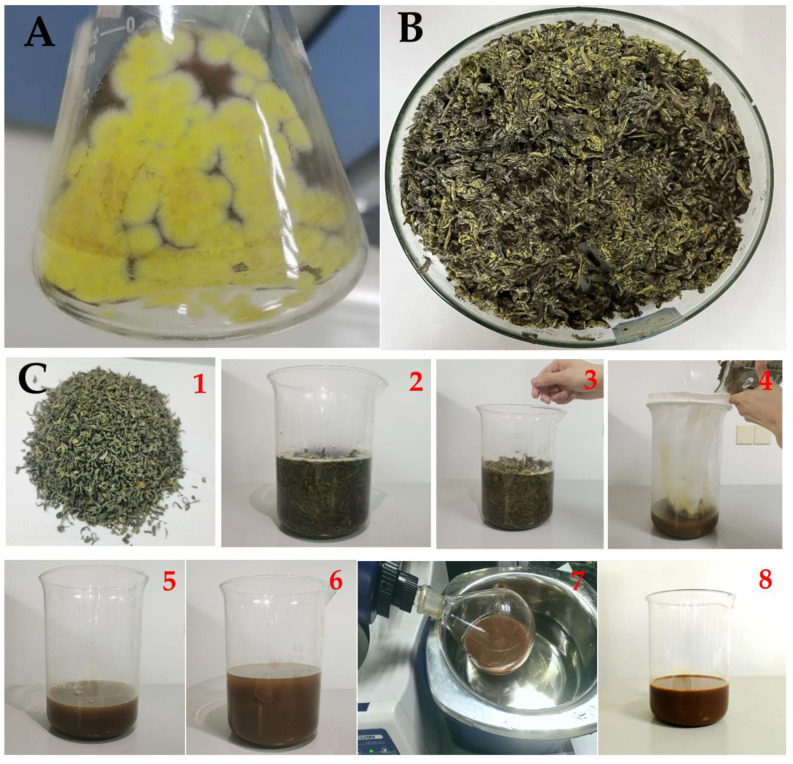
The processing diagram of making GTEG. (**A**) Green tea fermentation strain *Eurotium cristatum*; (**B**) Fermented green tea waste; (**C**) Process of making GTEG. It includes extraction, filtration, repeated extraction, decompression concentration and addition of MTG. In (**C**), (**C1**) Fermented green tea waste; (**C2,3**) Fermented green tea wastewater (GTW): Add hot water according to the ratio of material to liquid 1:12, keep it warm for 20 min at 75 °C to 80 °C. Stir three times for three minutes each time; (**C4**) 200 mesh filter; (**C5,6**) The filtered tea residue was extracted twice, and the ratio of solid to liquid was 1:7; (**C7**) Fermented green tea waste extract (GTE): Decompression and concentration. The concentration ends when the density is 1.20–1.21;(**C8**) Fermented green tea extract gels (GTEG): Add commercial MTG at a ratio of 1:1000.

Put 60 g of green tea waste into the fermentation box (the diameter of the fermentation box is 30 cm, the height is 5 cm), add 40 mL of sterile water to mix, and keep the water content of the solid fermentation system at about 40%. Inoculated with 15% suspension of M. coronoid, covered with plastic film, placed evenly in constant temperature and humidity incubator, and fermented at 30 °C for 7 days (Figure 1A,B). The fermented tea leaves were dried and stored at 4 °C.

Sun-dried fermented tea leaves were mixed with sterile water at a ratio of 1:12 (tea leaves: water) and kept warm at 75–80 °C for 20 min (Figure 1C). During this period, the tea leaves were stirred three times at 4-min intervals, and each round of stirring was performed for 3 min. After 20 min, the tea leaves were filtered through 200 mesh nets, and the filtered tea leaves were mixed with water at a ratio of 1:7 (tea leaves: water) for another round of extraction and filtering per the aforementioned method. The twice-filtered solution was concentrated under vacuum pressure at <0.07 mpa and 70 °C. After moisture level of <65%, and 200-mesh filtration were achieved, filtration samples were mixed commercial MTG at a ratio of 1:1000, the final sample stored at 0–4 °C in sterilized containers as crude liquor for the subsequent experiments.

### 2.4. FTIR Spectroscopy

FTIR analysis of the MTGase-induced GETG was performed by using a Nicolet 6700 FTIR spectrometer (Thermo Nicolet Co, Ltd., Madison, WI, USA). 1 mL of each sample was taken directly as measured on the attenuated total reflectance (ATR) accessory. The samples were measured by determining the ATR from 400 to 4000 cm^−1^ with a resolution of 4 cm^−1^ and a total of 64 scans. Changes from 500 to 4000 cm^−1^ were analyzed using Omnic software (version 6.0, Nicolet, Cheshire, UK) and PeakFit software (version 4.12, Systat Software, Richmond, CA, USA). The corresponding relationships between absorption peak and characteristics of the component were as follows: 1280–1290 cm^−1^ for tea polyphenols and caffeine; 1700–1780 cm^−1^ for amino acids, sugars or tea polyphenols; and 2900–2960 cm^−1^ for carbohydrates and proteins.

### 2.5. Determination of Rheological

Rheological was determined as described by Molina et al. [13]. with modifications, using a DHR-3 rheometer (TA Instruments, Leatherhead, UK) with parallel plates (1 mm gap and 40 mm diameter) in low-amplitude oscillatory frequency sweep mode. The frequency was modified from 0.1 to 10 Hz, and all experiments were performed at an invariable strain of 0.01%, which was within the linear viscoelastic region. The storage modulus (G′) and loss modulus (G″) were measured. All experiments were carried out at a constant temperature of 25 °C.

### 2.6. Determination of Gel Strength

Gel strength was determined as described by Qin et al. [14]. Samples of gels 10 mm in height and 20 mm in diameter were prepared by cutting them from the center of the gels. Using a TA-XT plus texture analyzer (Stable Micro Systems Co. Ltd., Godalming, UK), with a P/0.5-diameter probe connection. The samples were punctured at a constant speed of 5.0 mm s^−1^ to a maximum target depth of 10 mm. Gel strength was defined as the maximum squeeze stress required for puncture.

### 2.7. Determination of WHC

The gel samples (5 g) were transferred to 10 mL centrifuge tubes and centrifuged at 8000× *g* for 20 min at 4 °C in a CR22G II centrifuge (Hitachi Ltd., Tokyo, Japan). Reserve the centrifugal tube for pouring, and transfer the remaining water carefully with filter paper. After centrifugation, accurately weigh the water removed from the outer tube. The calculation formula for WHC is as follows:

WHC = ([Wt − Wr]/Wt) × 100%

where Wt is the total weight of water (g), Wr is the total weight of water removed (g).

### 2.8. Scanning Electron Microscopy (SEM) Analysis

The scan electron microscopy (SEM) images of GTEG, GTE and GTW were acquired using a scanning electron microscope (Quanta250FEG, FEI company, State of Oregon) operating at an accelerated voltage of 3.0 KV. A volume of 1 mL samples was lyophilized for 72 h, and each sample powder then was moved on a silica slice. The dried samples were sputter-coated with platinum for 2 min prior to imaging.

### 2.9. Determination of Physical and Chemical Constituents of GTEG

The tea polyphenol content of the GTEG was determined through the Folin’s reagent method. Specifically, 0.1 g of the tea polyphenol sample was placed in a beaker, 25 mL of water was added to dissolve the sample fully, and the sample was then cooled at a constant volume in a 100-mL volumetric bottle and subsequently used as the crude liquor. Next, 2.5 mL of the crude liquor was transferred to a 50-mL volumetric flask, 1 mL of the sample or standard product was mixed thoroughly with 5 mL of ferrous tartrate reagent, a phosphoric acid buffer of pH 7.5 was used to achieve a constant volume of 25 mL, and the absorbance value in a 1-cm colorimetric dish was measured at 540 nm. Thereafter, 1 mL of the sample or standard product was mixed with 5 mL of 10% Folin’s reagent, and 4 mL of 7.5% sodium carbonate solution was added within 3–8 min and mixed thoroughly with the solution; the resulting solution was allowed to react for 60 min, and its absorbance value was then measured at 765 nm. Gallic acid was the basis for the standard vertebral curve that was used to calculate the content of tea polyphenols.

### 2.10. Determination of Total Flavonoid Content

The aluminum trichloride colorimetric method was used for determining the total flavonoid content. Specifically, 0.5 mL of test solution was added to 10 mL of 1% aluminum trichloride solution, and the resulting solution was thoroughly stirred for 10 min; a 1% aluminum trichloride solution was used as the blank solution, and its absorbance was measured at 420 nm [15].

### 2.11. Determination of Soluble Sugar Content

The anthrone–sulfuric acid method was adopted for determining the soluble sugar content. After the GTEG was diluted 15 times, 1 mL of anthrone–sulfuric acid solution was mixed with 8 mL of anthrone–sulfuric acid solution, which was added to the GTEG solution, which was then bathed in boiling water for 7 min. The solution was immediately removed and cooled at room temperature in an ice water bath for 10 min. Soluble sugar concentration was measured using a standard curve of glucose.

### 2.12. Determination of Caffeine Content

A total of 20 mL of the test solution was accurately absorbed and transferred to a 250-mL volumetric flask. Next, 10 mL of 0.01 N hydrochloric acid and 2 mL of lead acetate solution were added, diluted to scale with water, fully mixed, and allowed to stand for clarification and filtration; 50 mL of accurately absorbed filtrate was injected into a 100-mL volumetric flask, 0.2 mL of 9 N sulfuric acid solution was then added, diluted to scale with water, thoroughly mixed, and allowed to stand for clarification and filtration. Absorbance was measured with a 10-mm colorimetric cup at 274 nm, with the blank solution of the reagent used as the reference, and the standard curve was used to measure the caffeine content of the solution.

### 2.13. Determination of DPPH Free Radical Scavenging Rate

A total of 0.2 mmol/L 2,2-diphenyl-1-picrylhydrazyl (DPPH) anhydrous ethanol solution was prepared. The DPPH solution was not added to a blank tube, and distilled water was used as the control; 2 L of each was added, mixed, and placed in the dark at room temperature for 30 min. Absorbance was measured at 517 nm. DPPH clearance rate was calculated using the formula as follows: (1 − [Sample − Blank]/Control) × 100%; three parallel tests were performed, and the results were averaged. The DPPH clearance rates of GTEGs with various concentrations were calculated.

### 2.14. Determination of ABTS Free Radical Scavenging Rate

In total, 3 mg of 2,2′-azino bis (3-ethylbenzothiazoline-6-sulfonic acid) (ABTS) and 1 mg of K_2_S_2_O_2_ were dissolved in 0.8 and 1.5 mL of distilled water, respectively, and shaken thoroughly. Next, 0.2 mL of each solution was mixed and dark oxidized for 12–16 h and diluted with methanol before use. An absorbance value of 0.7 ± 0.02 was obtained at 734 nm. Finally, 1 mL of liquor extract was added to 4 mL of the ABTS solution; the resulting solution was thoroughly mixed, allowed to react in the dark for 6 min, and adjusted to zero with ultrapure water; for reference, L-ascorbic acid was used as a positive control. The absorbance of the sample was measured at 734 nm and calculated using the formula for calculating DPPH clearance rate, which is described in the preceding paragraph. The results obtained were averaged for three parallel tests.

### 2.15. Animal Treatment

An amount of green tea polyphenols equivalent to 8–16 cups of green tea EGCG per day was used as the reference [16]. On the basis of the calculated tea polyphenol content in the GTEG crude liquor, the tea polyphenol dose in low-concentration GTEG was determined to be 200 mg/kg. Low-concentration GTEG was used as the standard, high-concentration GTEG was configured, and the tea polyphenol dose was controlled at 400 mg/kg. The final low-concentration GTEG configuration was 88 mg of tea extract crude liquor + 15 mL of ultrapure water, and the final high-concentration GTEG configuration was 176 mg of tea extract crude liquor + 15 mL of ultrapure water.

Twenty 6-week-old C57BL/6N male mice that weighed between 18 and 20 g were purchased from GemPharmatech. The mice were kept and fed in an environment with a temperature range of 22 ± 2 °C, a relative humidity of 50–60%, and a light/dark cycle of 12 h. All the mice were allowed to eat the standard pellet diet.

**Figure 2:**
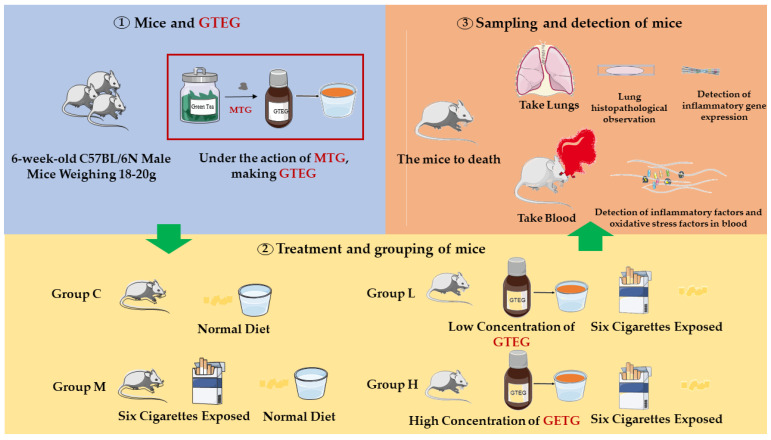
The processing diagram of this study. The main experimental steps were described, including preparation of mice and GTEG, grouping and treatment of mice, and testing of samples.

As indicated in Figure 2, the 20 C57BL/6 mice were randomly divided into four groups (*n* = 5), namely a natural control group (Group C), a passive smoking model group (Group M), a GTEG high-dose group (Group H), and a GTEG low-dose group (Group L). Mouse models were established through exposure to passive smoking. Except for the mice in Group C, the other mice were placed in the smoke machine to simulate artificial passive smoking. The mice were exposed to smoke twice a day over a period of 5 days; during each exposure session, six cigarettes were used with each accounting for 6 min of smoke production, and a smoking interval of 5 min was applied. The GTEG treatment groups were fed various concentrations of GTEG at 8:00 a.m. every day. Group H was fed 12 mg/mL GTEG, and Group L was fed 6 mg/mL GTEG; by contrast, Group C and Group M was only given distilled water.

### 2.16. Sample

After 5 days of experimental treatment, the mice fasted for 12 h before they were sacrificed. The mice were anesthetized through a peritoneal injection of pentobarbital sodium. The chest cavity of each mouse was opened, and the whole lung was removed. Each sample was cleaned with precooled 0.9% NaCl solution, and an RNA storage medium was added at a medium-to-solution ratio of 1:10. The samples were stored in formalin in a refrigerator at −80 °C for genetic analysis.

### 2.17. Monitoring the General Characteristics of Mice

The mice were fed at 8:00 a.m. daily, and the feeding standard was 15 g of feed per mouse for each day. The weight of each mouse was recorded after feeding. Unfinished feed was weighed at 8:00 a.m. the next day, and the amount that each mouse ate was recorded. In addition, the mental state, active state, hair color, and other attributes of the mice were observed and recorded during the daily feeding sessions.

### 2.18. Pulmonary Pathology of Mice

After the mice completed the experimental treatment, their whole lungs were removed (from their chest cavity), cleaned, and photographed. The complete lung leaf was observed to visually identify with the naked eye the external morphological changes in the lung tissue. The right upper lobe lung tissue of each mouse was soaked in formalin solution, and the pathological section of this lung tissue was analyzed. The remaining lung tissue was cryopreserved at −80 °C.

### 2.19. Detection of Serum Levels of T-SOD, MDA, GSH-Px, IL-6, and TNF-α in Mice

Blood was collected from the eyeballs of the mice, and the collected whole blood was stored in a 1.5-mL or 2-mL centrifuge tube, placed in a 4 °C refrigerator for 1 h, centrifuged twice at 4 °C for 3000 r/min for 15 min; thereafter, the resulting supernatant was collected per the manufacturer’s instructions for the test kit that was used. Changes in the expression levels of T-SOD, MDA, GSH-Px, IL-6, and TNF-α were detected.

### 2.20. Detection of Il-6, IL-33, and IL-1β Genes in Lung Tissue through Reverse Transcription–Polymerase Chain Reaction

A total of 1 mL of RNA extract was placed in a homogenate tube, which was then put on ice for precooling. Next, 100 mg of tissue was added to the homogenate tube and ground thoroughly until no tissue was visible. Total RNA was extracted from the lungs of the mice through the TRIzol method. RNA was reverse transcribed into cDNA by using the Servicebio RT First Strand cDNA Synthesis Kit. Reverse transcription–polymerase chain reaction (RT-PCR) was conducted per the manufacturer’s instructions for the SYBR Green qPCR Master Mix (no ROX) kit. The reverse transcription reaction system (20 μL) comprised the following: 4 μL of 5 × reaction buffer, 0.5 μL of Oligo (dT) 18 Primer (100 μM), 0.5 μL of Random Hexamer Primer (100 μM), 1 μL of Servicebio RT Enzyme Mix, 10 μL of total RNA, and 4 μL of nuclease-free water. Glyceraldehyde 3-phosphate dehydrogenase (i.e., GAPDH) was used as an internal reference, and the mRNA relative expression levels of the inflammatory genes related to IL-6, IL-33, TNF-α, and IL-1β were analyzed (Table 1 presents the primer sequences of each gene). RT-PCR amplification comprised three steps, namely denaturation at 95 °C for 15 s, annealing at 60 °C for 30 s (40 cycles with a 0.5 °C reduction for each cycle), and extension at 72 °C for 20 s (40 cycles). The calculation parameters and formulas pertaining to 2-δCT data processing are as follows: A = CT (target gene, sample to be tested) − CT (internal standard gene, sample to be tested), B = CT (target gene, control sample) − CT (internal standard gene, control sample), K = A − B; expression multiple = 2 − K.

### 2.21. Statistical Analysis

All experiments were repeated three times for each sample. The SPSS software version 19 (SPSS Inc., Chicago, IL, USA) was used for statistical analyses. An independent samples *t* test was performed to identify significant between-group differences. All data are expressed as means ± standard deviations (SDs). *p* < 0.05 was considered statistically significant.

## 3. Results

### 3.1. Gelation Properties of GTEG

**Figure 3:**
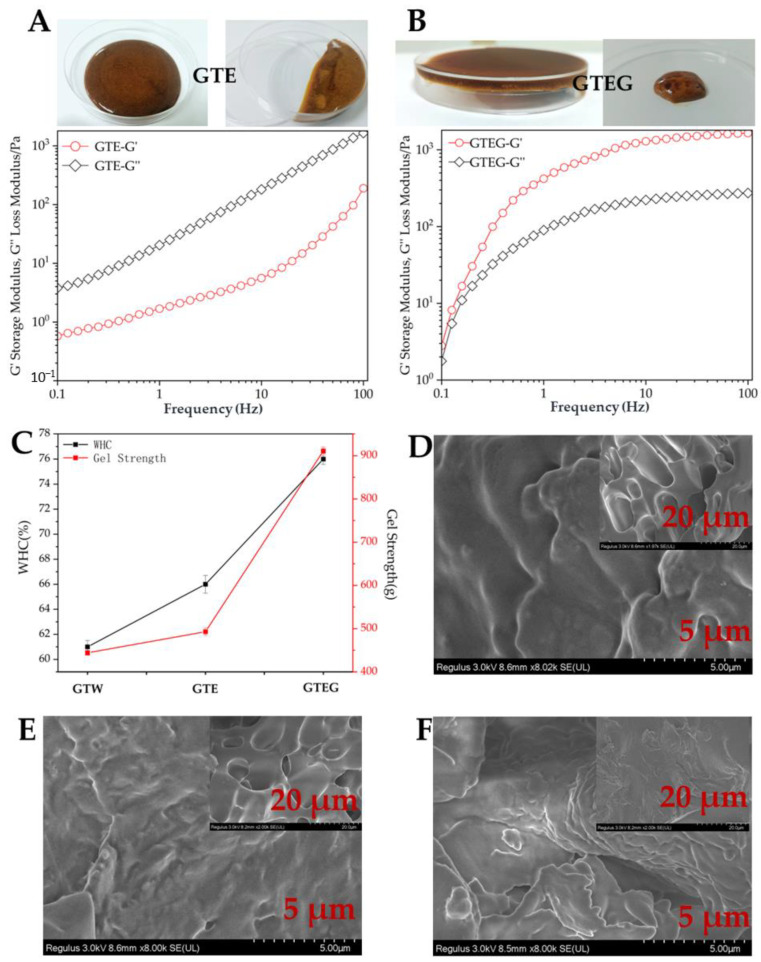
Gelation properties of GTEG. (**A**–**B**) Energy storage modulus G′ and loss modulus G″ as a function of angular frequency ω; (**C**) GTEG gel strength; (**D**–**F**) SEM images of GTW (**D**), GTE (**E**) and GTEG (**F**).

We studied the rheological properties of GTEG, checked its gel strength and analyzed it gel network by SEM. Figure 3A, B showed the energy storage modulus (G′) and loss modulus (G″) as a function of frequency. The results displayed that the storage modulus (G′) and loss modulus (G″) of GTEG and fermented green tea waste extract (GTE) increased with increasing frequency. But the difference is that the G′ value of GTEG is always greater than the G’’ value in the frequency range, while the G′ value of GTE is always less than G″. Moreover, G′ values of GTEG increased sharply from 0.1 to 1 Hz, then continued to increase slowly from 1 to 100 Hz, and exhibit nonterminal flow behavior (G′ > G″) in the experimentally achieved frequency range. The G′ values represent the elastic characteristics of gel samples or the solid-like behavior of the material, while the G″ values reflect the viscous characteristics of gel samples or the fluid-like behavior of the material. Thus, the data presented in Figure 3A, B indicated that MTGase-induced GTEG resulted in the formation of gels.

Gel strength and WHC are important characteristics of MTGase-induced gels. Therefore, we analyzed gel strength and WHC of fermented green tea wastewater (GTW), GTE and GTEG. As shown in Figure 3C, the gel strength of GTEG (911.98 g) is higher than that of GTW (444.03 g) and GTE (493.27 g). According to the results of Figure 3A,B, the characteristics of GTE tend to be viscosity and fluidity (G″ > G′), while GTEG reflects the elastic and solid-like characteristics of the gel. The results of Figure 3C further illustrate this conclusion. In addition, the results showed that the water holding capacity also increased with the increase of gel strength and presented a linear relationship (Figure 3C), which was also similar to the research results of Qin et al. [14].

Next, we observed the microstructure of GTW, GTE and GTEG by scanning electron microscope. The self-assembled fibrillar and porous structures formed at microscales within a diameter of 5 μm and 20 μm. Differences between the three samples are obvious. In the morphology of the GTEG, we can see the self-assembled fiber was shortest, and the network was the most compact. The GTEG induced by MTGase revealed good gel properties; this is shown in the morphological images and is supported by the rheological and WHC results (Figure 3A–C). Therefore, because of the aggregating behavior of the MTGase crosslinking interaction, GTE turns into GTEG with gel characteristics.

### 3.2. Physicochemical Constituents and Antioxidant Activity of GTEG In Vitro

**Figure 4:**
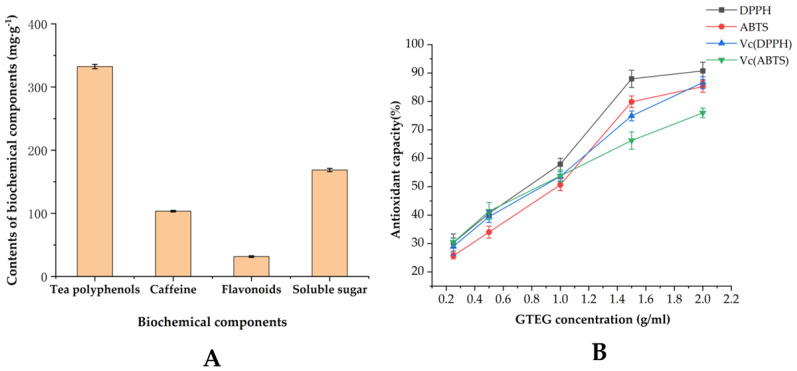
Chemical constituents and antioxidant capacity of GTEG. (**A**) Contents of tea polyphenols, caffeine, flavonoids, and soluble sugar in GTEG; (**B**) Antioxidant capacity of GTEG.

The GTEG was prepared through high temperature extraction, steam concentration, and various other steps. The experimental results revealed that the extraction rate of green tea was 58%; Figure 4A presents the analysis of its main physical and chemical components. The polyphenol, theine, total flavonoid, and soluble sugar content levels of the GTEG were 332.43, 103.44, 31.54, and 168.65 mg/g, respectively. The antioxidant activity of the GTEG in vitro was also analyzed; Figure 4B presents the analysis results, which indicated that when the concentration level was between 0.25 and 1.5 g/mL, the DPPH and ABTS free radical scavenging rates of the GTEG increased significantly with an increase in concentration. When the concentration level of the GTEG was >1.5 g/mL, its antioxidant level was stable and responded gradually to changes in concentration.

### 3.3. FTIR Analysis of GTEG

**Figure 5:**
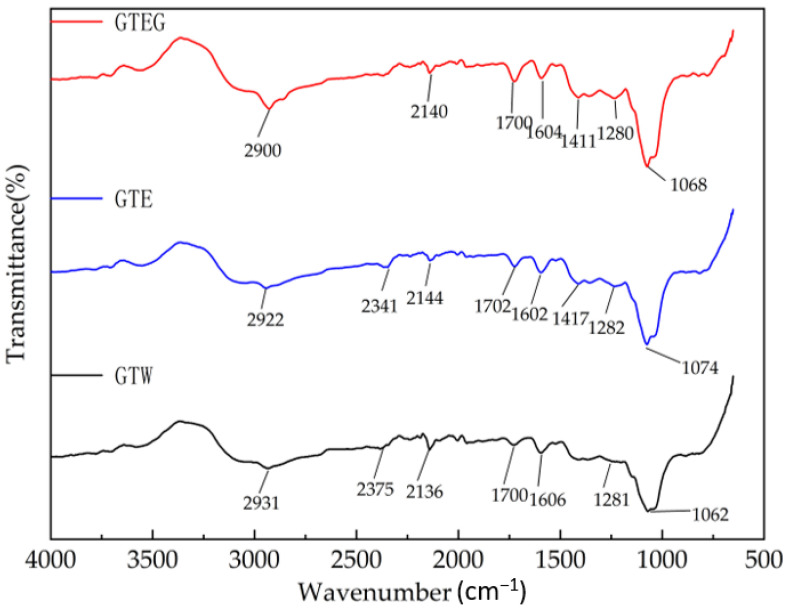
FTIR of green tea at different treatment stages. GTEG: green tea waste extract gels; GTE: green tea waste extract; GTW: green tea wastewater.

The spectral changes of phenols, sugars, and caffeine in GTEG, GTE and GTW were determined by Fourier infrared spectroscopy (FTIR). The peak of 1280–1290 cm^−1^ is C-OH and C-N stretching vibration, and the source compounds are mainly tea polyphenols and caffeine. The 1700–1780 cm^−1^ peak was the stretch vibration site of C=O and C=C, and the source compounds were amino acids, sugars and tea polyphenols. The absorption peaks near 2900–2960 cm^−1^ are mainly due to superimposed absorption of hydroxyl and amino groups in carbohydrates and proteins. Figure 5 result shows FTIR (400–4000 cm^−1^) of GTEG, GTE and GTW. Compared with GTE and GTW, GTEG was stronger at the absorption peaks of 1700 cm^−1^ and 2900 cm^−1^, and medium at 1280 cm^−1^ (Figure 5). The corresponding peaks of GTE are 1282 cm^−1^ (tea polyphenols and caffeine), 1702 cm^−1^ (amino acids, sugars and tea polyphenols) and 2922 cm^−1^ (carbohydrates and proteins) respectively, and the corresponding peaks of GTW are 1281 cm^−1^ (tea polyphenols and caffeine), 1700 cm^−1^ (amino acids, sugars and tea polyphenols), 2931 cm^−1^ (carbohydrates and proteins), respectively. Compared with GTEG, the peak patterns of GTE and GTW are similar, but some of the peaks move forward and the intensity changes to some extent; it indicates that the contents of tea polyphenols, caffeine, sugar and other substances in GTEG change during the production process (Figure 5).

### 3.4. Effects of CS on General Characteristics of Mice

**Figure 6:**
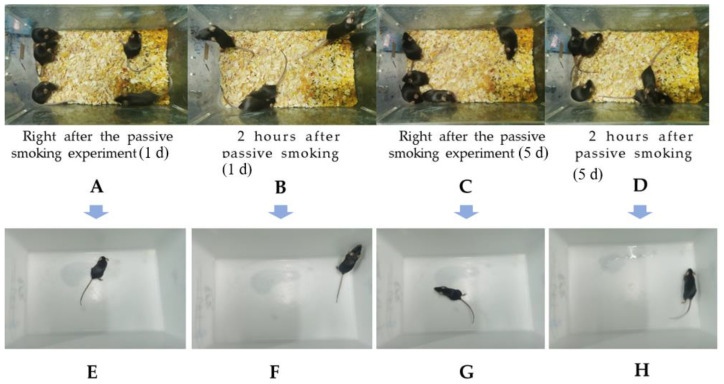
Effects of passive smoking on mental state of M group mice (**A**,**B**) Mental state of the mice on the first day of the experiment; (**C**,**D**) The mental state of mice on the fifth day of the experiment; (**E**–**H**) Mice from (**A**–**D**) were randomly selected for separate observation.

In accordance with the experimental design, the mice in Groups H and L were treated with GTEG, and those in Groups M, H, and L were exposed to CS; the mental state, active state, hair color, and other indicators of the mice were monitored during the experiment. The experimental results indicated (Figure 6) that the mice responded actively to smoke exposure during the early stage of passive smoking; they exhibited actions that indicated that they wanted to escape the smoke during the process of passive smoking (Figure 6A). After each session of passive smoking, the mice became highly active and aroused (Figure 6B). During the later stage of passive smoking modeling, they exhibited slow reaction to external stimuli, mental fatigue, reduced activity, and rough and dull hair (Figure 6C,D).

**Figure 7:**
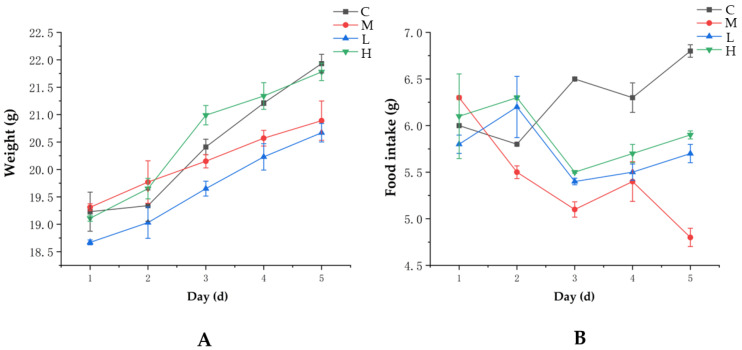
Effects of passive smoking on body weight and food intake in mice (**A**) Changes in body weight of mice during the experiment; (**B**) Changes in food intake in mice during the experiment.

In addition, the body weight and food intake of the mice were measured; the results indicated that compared with Group C (i.e., natural control group), the final weight gain in the Group M (i.e., passive smoking model group) mice was smaller by 1.12 g, whereas the weight gain results of the GTEG-treated mice (i.e., Groups H and L) indicated a recovery. The final weight gain in Group H was 1.11 g more than that in Group L; however, the mice in Group H still did not fully regain their weight relative to the growth level of the mice in Group C (Figure 7A). The changes in the feed intake of the mice revealed that the feed intake of the mice in Group M fluctuated but exhibited an overall downward trend, whereas the feed intake of the mice in Groups L and H increased during the first 3 days of the experiment and exhibited an upward trend (Figure 7B). These results suggest that an increase in GTEG treatment time helped the mice to gradually recover in terms of weight and food intake.

### 3.5. Effects of GTEG Treatment on Lung Tissue of Passive Smoking Mice

**Figure 8:**
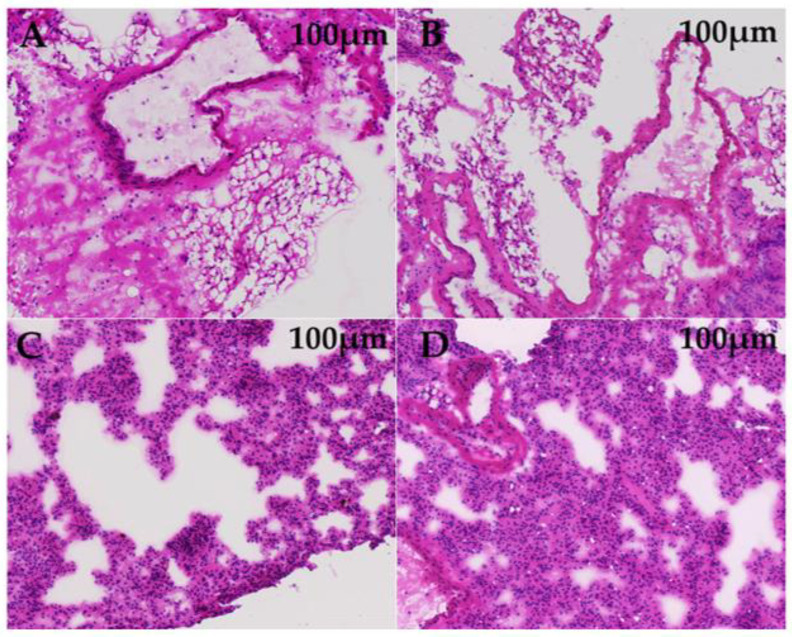
H&E staining of lung sections. (**A**) Control group; (**B**) Model group; (**C**) GTEG low-dose group; (**D**) GTEG high-dose group.

To explore the effect of GTEG treatment on the lung tissue of passive smoking mice, we conducted pathological analyses of the lung tissue of the mice from each group. Under a light microscope, the following results were revealed. The lung tissue of the mice in Group C exhibited a complete alveolar structure with a regular and uniform shape, and the microvessels in the examined alveoli were complete and did not exhibit bleeding or tissue fluid exudation (Figure 8A). The alveoli of the Group M mice were enlarged and irregular in shape, with septal fracture and severe damage to the tissue structure (Figure 8B). The alveolar cavities of the Group L mice were materialized, and almost no alveoli were observed; hyperplasia of the epithelial tissue and the infiltration of numerous inflammatory cells were also observed (Figure 8C). The Group H mice had irregular alveoli, and some alveolar cavities were materialized with inflammatory cell infiltration (Figure 8D). Although the pathological changes in the lung tissues from Groups L and H were more severe than those in the lung tissues from Group C, the inflammation experienced by the mice in Groups L and H was reduced compared with that experienced by the mice in Group M. Pathological section analysis revealed that passive smoking damaged the lung tissue of mice and that the GTEG treatment promoted the repair of lung tissue.

### 3.6. Effect of GTEG on the Expression Level of Oxidative Stress Factor in Serum of Mice

To explore the effect of the GTEG on the oxidative damage response of mice, we analyzed the serum oxidative stress factors of mice and detected T-SOD activity, MAD content, and GPH-Px activity. Table 2 reveals that compared with the Group C mice, the T-SOD activity of the Group M mice decreased by 21.45%; however, compared with the Group M mice, the mice in Groups H and L exhibited increased T-SOD activity after receiving the GTEG treatment, and this increase was more significant in Group H (*p* < 0.05). The serum MAD content in the Group M mice was significantly increased compared with that in the Group C mice (*p* < 0.05), whereas the serum MAD content in Groups L and H was significantly decreased (41.92% and 43.64% for Groups L and H, respectively) compared with that in the Group M mice (*p* < 0.05). Compared with the serum GPX activity of Group C mice, the serum GPX activity of the mice in Groups M, H, and L (cigarette treatment groups) was significantly decreased (*p* < 0.05); however, a gradual recovery to normal levels was achieved by the mice in Groups H and L (i.e., GTEG treatment groups).

### 3.7. Effect of GTEG on the Expression Level of Oxidative Stress Factor in Serum of Mice

**Figure 9:**
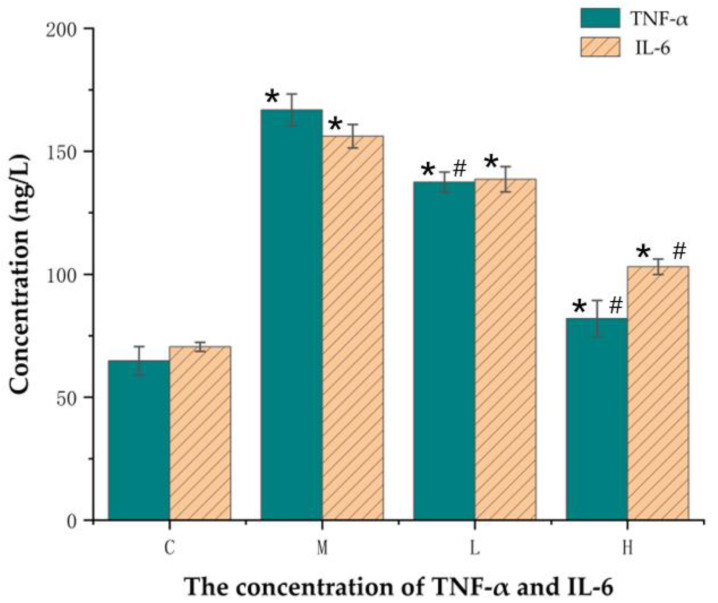
Changes of serum cytokine expression in mice. * Compared with C group, # Compared with M group, *p* < 0.05 showed a significant difference.

We investigated the systemic inflammatory cytokines that were present in mouse serum. Among them, tumor necrosis factor TNF-α produces a strong antiviral effect by activating the expression of IL-6, a cytokine essential for acute inflammatory responses. Figure 8 reveals that compared with the Group C mice, the concentrations of TNF-α and IL-6 in the Group M mice were increased by 139.99% and 157.43%, respectively, indicating that CS treatment activated the inflammatory response system of the Group M mice in vivo (Figure 9); however, after treatment with the GTEG, the concentrations of TNF-α and IL-6 in the mice were decreased to varying degrees; notably, the concentrations of TNF-α and IL-6 in the Group H mice were decreased to 93.065 and 82.006 ng/L, respectively, which were similar to the levels observed in Group C and indicated that the GTEG treatment alleviated inflammation to an extent. The aforementioned effect was more significant (*p* < 0.05) in the Group H mice (high-concentration treatment group) than in the Group L mice (low-concentration treatment group).

### 3.8. Effects of GTEG on the Expression of Pulmonary Inflammatory Genes in Mice

**Figure 10:**
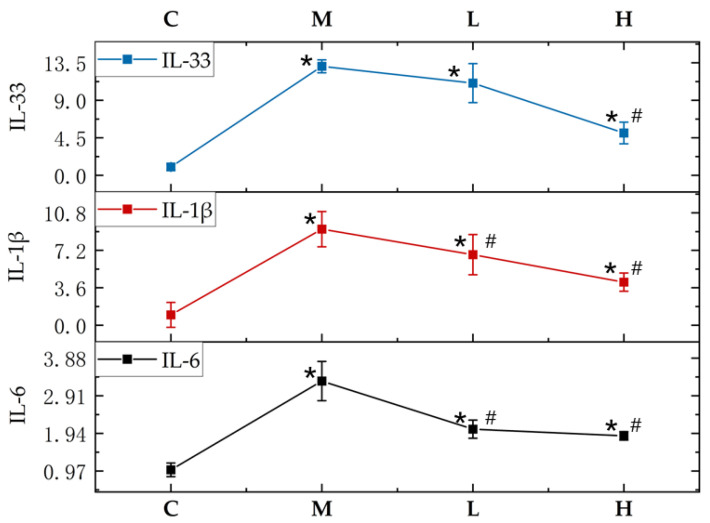
Changes in lung inflammation gene expression in mice. The blue line represents the relative expression multiple of IL-33. The red line shows the relative expression multiple of IL-1β. The black line shows the relative expression multiples of IL-6. * Compared with C group, # Compared with M group, *p* < 0.05 showed a significant difference.

The expression levels of IL-33, IL-β, and IL-6 genes in lung tissue were detected; compared with the group C mice, the expression levels of IL-33, IL-β, and IL-6 genes were significantly increased in the Group M mice (*p* < 0.05; Figure 10), indicating that passive smoking exacerbated lung inflammation symptoms; however, compared with the Group M mice, the expression levels of IL-33, IL-β, and IL-6 genes were significantly decreased in the mice in Groups H and L after the GTEG treatment; this decrease was more prominent in Group H (i.e., the high-concentration treatment group) than in Group L (i.e., the low-concentration treatment group), indicating that the GTEG reduced the expression of inflammatory genes and the inflammatory response of the examined mice.

## 4. Discussion

CS pollutes the environment and seriously harms human health and quality of life. Long-term exposure to harmful gases and particulate matter can induce oxidative stress, inflammation, imbalance of protease and antiprotease in the airway and lungs, and even chronic continuous airflow restriction [17], which is a considerable threat to human health. CS contains more than 5000 chemical substances, and each mouthful of CS can directly or indirectly generate more than 1015 units of active aldehyde, benzopyrene, quinine, and other oxidants in the human body through the Haber–Weiss reaction or other mechanisms, and all of them can cause lung diseases [18]. In the present experiment, passive smoking led to alveolar enlargement, irregular shaped alveoli, septal rupture, and severe tissue structure damage in mice; these findings are similar to those reported by Li et al. [19]; however, the level of inflammation in the mice was reduced after they received the GTEG treatment.

Green tea is beneficial for individuals with various chronic diseases because of its strong antioxidant activity [20]. Tea polyphenols are the main active ingredients of tea, which mainly comprise eight catechins; these catechins exhibit high levels of EGCG content and biological activity and have been extensively studied because of their strong antioxidant properties [21]. The flavonoids in tea exhibit a strong anti-inflammatory effect and antibacterial activity [22], and caffeine, which is also a bioactive substance in tea, exerts cardiac strengthening, excitement, and diuretic effects [23]. In this experiment, GTEG was induced by MTGase. MTGase is an enzyme that action between glutamine and lysine protein led to a cross-linking and polymerizing effect by ε-(γ-glutamyl) lysine bonds. Through rheological and SEM studies, GTEG has more gelation properties than GTE and GTW, which may be because MTGase covalently cross-linked molecules in GTE to form GTEG. Gel strength and WHC are the most important characteristics of MTGase-induced gels [14], the linear relationship between the water holding capacity of GTEG and gel strength further indicated that molecular crosslinking occurred in GTEG. Next, to clarify the material basis of the biological activity of the GTEG, we measured the physical and chemical components of tea polyphenols, flavonoids, and caffeine in the GTEG. The results indicated that the composition of physical and chemical components in the GTEG was similar to that in green tea. The GTEG contained up to 332.43 mg/g of tea polyphenols, and the ratio of soluble sugar and flavonoids also indicated that the GTEG preparation process allowed for the physical and chemical compositions of the original green tea to be retained. On the basis of the results pertaining to physical and chemical components, we further analyzed the antioxidant activity of the GTEG in vitro to preliminarily clarify the biological activity of the GTEG [24]. In the present study, the antioxidant capacity of the GTEG in vitro was characterized by the scavenging ability of DPPH and ABTS free radicals. The results indicated that the antioxidant capacity of the GTEG increased when its concentration increased; notably, when its concentration was >1.5 g/mL, its antioxidant level was stable and responded gradually to changes in concentration, reaching 80–90%; this result is similar to that reported by Thring et al., who also studied the antioxidant effects of green tea [20]. The high antioxidant activity of the GTEG in vitro also provided a preliminary basis for the subsequent experiment in which the GTEG was used to reduce the oxidative stress induced by CS in vivo.

Next, we studied the effect of the GTEG on the oxidative stress response of mice that were exposed to CS. Under normal physiological conditions, oxidation and antioxidation are dynamically balanced in the body, and imbalances tend to result in oxidation, that is, oxidative stress. Superoxide dismutase (SOD) is a key member of the antioxidant enzyme system in the body, and it can catalyze superoxide anion disproportionation to produce H2O2 and oxygen, which play crucial roles in maintaining the oxidation and antioxidant processes of the body [25]. MDA is one of the final products produced when reactive oxygen species attack membrane lipids and exhibits cytotoxicity. GSH-Px is a key peroxidase decomposition enzyme that widely exists in the body, and it is also an indicator of the body’s antiperoxidation ability [26]. In the present study, the T-SOD, MAD, and GSH-Px levels in the serum of the experimental mice were examined to determine the level of oxidative stress in the mice. The results revealed that the activity levels of T-SOD and GSH-Px in the serum of the passive smoking mice decreased significantly (*p* < 0.05) and that their MAD content increased significantly (*p* < 0.05), indicating obvious oxidative stress reactions in the passive smoking mice. The GTEG treatment significantly (*p* < 0.05) reduced the MDA content and increased the SOD and GSH-Px activity levels in the mice, that is, the level of oxidative stress in the mice was reduced. In addition, the results highlighted that a high concentration of the GTEG led to more favorable results relative to a low concentration of the GTEG; this finding suggests that the GTEG alleviated oxidative damage in the mice by reducing their peroxide levels and improving their endogenous antioxidant capacity; however, these effects differed depending on the GTEG concentration that was applied.

Inflammatory factor networks play crucial roles in maintaining and amplifying lung inflammation, and the fibrosis of small airways, damage of alveolar walls, and excessive secretion of mucus are directly caused by these intricate inflammatory networks [27]. Il-6 and TNF-α are key nodes in an inflammatory cytokine network, and their levels are considerably increased when severe lung inflammation occurs. Excessive IL-6 and TNF-α levels activate the NF-κB complex, which in turn promotes the expression of more inflammatory genes and amplifies the inflammatory response [27]. In addition, IL-6 and TNF-α are considered crucial systemic inflammatory factors causing chronic obstructive pulmonary disease complications such as osteoporosis, endothelial cell injury, and depression [28]. In the present experiment, we analyzed the contents of these two inflammatory factors in the serum of mice; the results revealed that compared with Group C, the levels of IL-6 and TNF-α in Group M were significantly (*p* < 0.05) increased by 139.99% and 157.43%, respectively, indicating that the modeling successfully induced an obvious inflammatory response. Our results are similar to those of Ying et al. and Nemmar et al., who also reported significantly elevated levels of IL-6 and TNF-α after CS treatment [29,30]. According to the experimental results, the concentration of IL-6 and TNF-α decreased after the GTEG treatment; this result suggests that the GTEG reduced the lung injury that was induced through passive smoking in the mice by reducing inflammation levels and through the antioxidant effect.

IL-33 is a member of the IL-1 family, which includes IL-1α, IL-1β, IL-1 receptor antagonist, and IL-18. Il-33 is mainly present in the skin, lungs, adipocytes, and synovial fibroblasts, and its biological effects are mediated through the T1/ST2 binding activation of NF-κB and MAP kinases [31]. Il-6 was originally identified as a B-cell stimulator that regulates various biological functions in a variety of physiological processes. Il-6 is a key member of the cytokine network; it plays a crucial role in acute inflammation and is associated with innate acquired immune responses [32]. In the present study, the mRNA expression levels of IL-33 and the inflammatory factors IL-6 and IL-1β were detected; the results indicated that IL-33, IL-6, and IL-1β were highly expressed in the mice from the pure smoking group; this finding suggests that when inflammation occurs because of chronic lung injury, lung cells may undergo severe apoptosis and release IL-33. IL-1β is a cytokine that predicts inflammation [33], and the expression level of IL-1β increases considerably when various infectious diseases occur; in particular, the lung injury caused by smoking in the present experiment caused the expression level of IL-1β to increase by 10 times. Il-6 is a crucial inflammatory cytokine that induces acute responses and cell proliferation and differentiation, and it can promote muscle catabolic metabolism by activating proteolysis. In Group M, the expression levels of IL-33, IL-6, and IL-1β was increased; however, when the mice were treated with various concentrations of the GTEG, the expression levels of IL-33, IL-6, and IL-1β were decreased, indicating that the GTEG reduced inflammation and oxidative stress in vivo in the passive smoking mice.

In conclusion, CS contains numerous harmful substances, and the passive inhalation of CS causes oxidative stress in the body. The results of the present study indicate that the GTEG can be used as an intervention to reduce the level of oxidative damage in vivo, which is achieved through alterations in the levels of oxidative stress factors and inflammatory factors in vivo and the expression levels of inflammatory genes. The present study also provides a basis for further research on the antioxidant and anti-inflammatory effects of the GTEG.

## Figures and Tables

**Table 1:** Gene primer sequence table.

Primer Information	The Name of the Primer	Primer Sequence (5′–3′)	Segment Length (BP)
NM_031168.2	M-IL6-S	TTCTTGGGACTGATGCTGGTG	177
	M-IL6-A	GCCATTGCACAACTCTTTTCTC	
NM_001164724.2	M-IL33-S	CAAAGTTCAGCAGCACCGC	278
	M-IL33-A	TGTGTCAACAGACGCAGCAAA	
NM_008361.4	M-IL1β-S	GCATCCAGCTTCAAATCTCGC	256
	M-IL1β-A	TGTTCATCTCGGAGCCTGTAGTG	
NM_008084.2	M-GAPDH-S	CCTCGTCCCGTAGACAAAATG	133
	M-GAPDH-A	TGAGGTCAATGAAGGGGTCGT	

NM_031168.2 is primer for IL-6; NM_001164724.2 is primer for IL-33; NM_008361.4 is primer for IL-1β; NM_008084.2 is primer for GAPDH.

**Table 2:** Changes of activity and content of oxidative stress factors in the serum of mice.

Groups	T-SOD Activity (U/mL)	Concentration of MAD (nmol/mL)	GSH-PX Activity (U/mL)
C	287.468 ± 7.355	10.025 ± 0.446	163.641 ± 2.462
M	225.779 ± 5.738 *	21.049 ± 1.018 *	100.225 ± 2.766 *
L	226.769 ± 3.488 *	12.225 ± 0.526 *#	130.808 ± 2.215 *#
H	240.464 ± 7.160 *#	11.863 ± 0.216 #	144.865 ± 6.763 #

* Compared with C group, # Compared with M group, *p* < 0.05 showed a significant difference.

## Data Availability

Data available on request from the authors.
